# Serial Low Doses of Sorafenib Enhance Therapeutic Efficacy of Adoptive T Cell Therapy in a Murine Model by Improving Tumor Microenvironment

**DOI:** 10.1371/journal.pone.0109992

**Published:** 2014-10-15

**Authors:** Hui-Yen Chuang, Ya-Fang Chang, Ren-Shyan Liu, Jeng-Jong Hwang

**Affiliations:** 1 Department of Biomedical Imaging and Radiological Sciences, National Yang-Ming University, Taipei, Taiwan; 2 National PET/Cyclotron Center and Department of Nuclear Medicine, Taipei Veterans General Hospital, Taipei, Taiwan; Institut Jacques Monod, France

## Abstract

Requirements of large numbers of transferred T cells and various immunosuppressive factors and cells in the tumor microenvironment limit the applications of adoptive T cells therapy (ACT) in clinic. Accumulating evidences show that chemotherapeutic drugs could act as immune supportive instead of immunosuppressive agents when proper dosage is used, and combined with immunotherapy often results in better treatment outcomes than monotherapy. Controversial immunomodulation effects of sorafenib, a multi-kinases inhibitor, at high and low doses have been reported in several types of cancer. However, what is the range of the low-dose sorafenib will influence the host immunity and responses of ACT is still ambiguous. Here we used a well-established E.G7/OT-1 murine model to understand the effects of serial low doses of sorafenib on both tumor microenvironment and transferred CD8+ T cells and the underlying mechanisms. Sorafenib lowered the expressions of immunosuppressive factors, and enhanced functions and migrations of transferred CD8+ T cells through inhibition of STAT3 and other immunosuppressive factors. CD8+ T cells were transduced with granzyme B promoter for driving imaging reporters to visualize the activation and distribution of transferred CD8+ T cells prior to adoptive transfer. Better activations of CD8+ T cells and tumor inhibitions were found in the combinational group compared with CD8+ T cells or sorafenib alone groups. Not only immunosuppressive factors but myeloid derived suppressive cells (MDSCs) and regulatory T cells (Tregs) were decreased in sorafenib-treated group, indicating that augmentation of tumor inhibition and function of CD8+ T cells by serial low doses of sorafenib were *via* reversing the immunosuppressive microenvironment. These results revealed that the tumor inhibitions of sorafenib not only through eradicating tumor cells but modifying tumor microenvironment, which helps outcomes of ACT significantly.

## Introduction

Adoptive T cell therapy (ACT) is considered to be a promising therapeutic strategy for cancer treatment. However, the clinical outcome and application of ACT are impeded by the requirement of large amounts of tumor-specific CD8+ T cells for infusion, anergy or apoptosis of transferred CD8+ T cells, and inadequate numbers of tumor-infiltrating CD8+ T cells [1]. Increasing evidences show that immunosuppressive networks in the tumor microenvironment act as substantial barriers to T cell response. Cancer cells hinder T cells responses through several pathways, including impairing antigen presentation, activating negative co-stimulatory signals (CTLA-4/B7 and PD-1/PD-L1), secreting immunosuppressive factors (i.e. transforming frowth factor-β (TGF-β) and interleukin-10 (IL-10)) to recruit immunosuppressive cells like regulatory T cells (Tregs), tumor-associated macrophages (TAMs) and myeloid-derived suppressor cells (MDSCs), and triggering proapoptotic pathways (indoleamine 2,3-dioxygenase (IDO), Fas ligand (FasL) and TNF-related apoptosis-inducing ligand (TRAIL)) in effector T cells [2], [3]. Because of high heterogeneity and dynamics of cancer, combination therapies are thought to be more efficient for cancer treatment than monotherapy [4]–[6]. However, combination of ACT and chemotherapy was reported as incompatible due to the induction of lymphopenia and immunosuppressive cytokines, and the inhibition of effector T cell function by chemotherapy at first [7]. Nevertheless, later reports show that preconditioning radiotherapy or chemotherapy performed before CD8+ T cell transfer modifies unfavorable tumor microenvironments [8], and improve the effectiveness of T cell therapy through inducing tumor cell deaths, eliminating Tregs, and enhancing tumor cell killing by effector T cells [9], [10].

Sorafenib, a multi- kinase inhibitor (TKI), blocks cancer progression through inhibiting RAS-RAF-MEK-ERK-MAPK pathway, which relates to the proliferation, migration and angiogenesis of cancer cells by targeting vascular endothelial growth factor receptor (VEGFR), platelet-derived growth factor receptor (PDGFR), c-kit and a receptor tyrosine kinase called rearranged during transfection (RET) [11]. Different from sunitinib, sorafenib has been reported that affects maturation and functions of dendritic cells (DCs), reduces the T cells responses, and is inappropriate for combination with immunotherapies [12]. On the other hand, several groups showed that sorafenib depletes frequencies of Tregs in RCC patients [13], [14]. Sorafenib also has been shown with higher cytotoxicity to CD4^+^CD25^+^ regulatory/suppressor T cells as compared with CD4+ or CD8+ T cells, indicating that sorafenib could eliminate Tregs without harming effector T cells [15], [16].

The underlying immunomodulation mechanisms of sorafenib and whether sorafenib could augment treatment outcomes of immunotherapies as ACT are still unclear. A well-established E.G7/OT-1 murine model combined with the *pGBeLT* imaging system to monitor the activation and distribution of CD8+ T cells after serial low doses of sorafenib treatments was used for therapeutic efficacy evaluation of ACT [17]. In brief, level of granzyme B increases when T cells are activated, thus, using granzyme B promoter to drive downstream imaging reporters may represent the activation of T cells. The results show that serial low doses of sorafenib enhances therapeutic responses of ACT significantly without harming the normal immunity. Sorafenib lowers the expressions of immunosuppressive proteins (e.g. TGF-β, IL-10 and VEGF) then augments the function and migration of CD8+ T cells both *in vitro* and *in vivo*. Moreover, serial low doses of sorafenib (7.5 mg/kg/day) reduce the percentages of Tregs and MDSCs, increase the numbers of activating T cells, and prolong the period of T cells activation. Our results suggest that the low doses of sorafenib augment ACT through reversing immunosuppressive tumor microenvironment and enhancing the functions of transferred CD8+ T cells.

## Materials and Methods

### Cell culture and reagents

OVA-expressing E.G7 mouse lymphoma cell line was obtained from ATCC, and maintained in cRPMI-1640 (Hyclone, Logan, UT) supplemented with 10% FBS (Hyclone), 1% penicillin/streptomycin (Gibco, Grand Island, NY), and 25 mM HEPES (Gibco). HEK-293FT cell line was purchased from Invitrogen and maintained in DMEM supplemented with 10% FBS, 1% penicillin/streptomycin, 2 mM L-glutamine (Gibco), and 0.1 mM MEM non-essential amino acids (Gibco). 400 and 500 µg/ml G418 (Calbiochem, La Jolla, CA) were also supplemented in the culture of E.G7 and HEK-293FT cells, respectively. Lentiviral production and titration assessment were performed as previously described [17].

For *in vitro* studies, sorafenib was extracted from tablets (Bayer HealthCare Pharmaceuticals, West Haven, CT) as previously described [18]. Both sorafenib and STAT3 inhibitor III, WP1066 (Calbiochem), were dissolved in DMSO at 10 mM and stored at −20°C. For *in vivo* studies, a tablet of sorafenib was dissolved in Cremophor EL (Sigma, St. Louis, MO) and 95% ethanol (1: 1) at the stock concentration of 5 mg/ml, and stored at −20°C [19].

### Ethics statement of animal work

The animal study was carried out in strict accordance with the recommendations in the Guide for the Care and Use of Laboratory Animals of the National Laboratory Animal Center. The protocol was approved by the Institutional Animal Care and Use Committee of National Yang-Ming University, Taiwan. (Permits Number: 1021267). 8-week-old male C57BL/6 mice were purchased from the National Laboratory Animal Center (Taipei, Taiwan), and OT-1 transgenic mice obtained from the Jackson Laboratory were housed in the B.S. animal room with 12 hours light/dark cycle of the Laboratory Animal Center of National Yang-Ming University. The imaging studies were performed under 2% isoflurane was used for anesthesia during tumor inoculation and imaging acquisition. CO_2_ was use for euthanasia the mice when isolated T cells or at the end of the experiment, and all efforts were made to minimize suffering.

### Cytotoxicity of sorafenib

E.G7 cells were treated with various concentrations of sorafenib for 24 hours, and AlamarBlue assay was used to assay the cytotoxicity of sorafenib. Briefly, 20 µl (1/10 volume of medium) of alamarBlue reagent (AbD Serotec, Oxford, UK) was added into cell culture and incubated at 37°C for 4 hours. The fluorescent signals were assessed at 570 and 600 nm, respectively, by ELISA reader (TECAN Sunrise, Zürich, Switzerland). The cell viability was calculated using the equation provided by the manufacture.

### Isolation and transduction of OT-1 CD8+ T cells

Splenocytes from 12 to 14-week-old OT-1 mice were enriched by using a mouse T cell enrichment kit (Stemcell Technologies, Vancouver, Canada), and cultured in cRPMI-1640 containing 2.5 µg/ml Concanavalin A (Calbiochem), 50 µM β-mercaptoethanol (Bio-Rad, Richmond, CA), 10 ng/ml IL-7 (R & D Systems, Minneapolis, MN) for 48 hours as previously described [17]. CD8+ T cells were transduced with pGBeLT lentiviruses (MOI  = 5) for 4 hours, and cultured in cRPMI-1640 containing 50 µM β-ME, 10 ng/ml IL-15, 10 U/ml IL-2 (R & D Systems), and 50 µM α-methyl mannoside (Calbiochem) for another 24 hours.

### ^51^Cr-release assay

1×10^6^ E.G7 cells pretreated with or without 5 µM sorafenib were resuspended in 500 µl cRPMI-1640 and incubated with 1.11×10^7^ Bq ^51^Cr (American Radiolabeled Chemicals, St. Louis, MO) for 2 hours. Then plated into U-bottom 96-well plates at the density 1×10^4^ cells/well and co-cultured with CD8+ T cells at various effector-to-target ratios for another 4 hours. The supernatant was collected and counted by the gamma counter (Wallac 1470 WIZARD Automatic Gamma Counter, Perkin-Elmer Life Science, Boston, MA). The percentage of specific lysis was calculated as follows: (experimental release - spontaneous release)/(maximum release - spontaneous release) ×100%. Maximum release was determined by measuring the counts of labeled E.G7 cells lysed with 1% Triton X-100.

### Transwell assay

1×10^6^/well E.G7 cells pretreated without or with 5 µM sorafenib or WP1066 were seeded into the 24-well plates. CD8+ T cells were resuspended at the density of 1.5×10^6^ cells/ml, and 50 µl of cell suspensions were added into the top of 5 µm transwell inserts (Corning Costar, Cambridge, MA). After 4 hours incubation, the membranes were fixed, stained with hematoxylin and air-dried. The number of migratory CD8+ T cells was counted under Leica DM IRB microscope (Deerfield, IL) with 100× magnification, and four fields per slide were imaged.

### Western blotting

Cell lysates were separated by SDS-PAGE, transferred to polyvinylidene difluoride membranes. Membranes were incubated with antibodies against IDO (Millipore, Bedford, MA), TGF-β, STAT3, phospho-STAT3, CCL2/MCP-1 (Cell Signaling, Beverly, MA), IL-10, VEGF (Abcam, Cambridge, MA), and β-actin (Novus, Littleton, CO) at 4°C overnight after blocked with 5% nonfat milk. After washing with Tris-Tween buffer saline, membranes were incubated with horseradish peroxidase-conjugated secondary antibodies (Jackson ImmunoResearch Laboratories, West Grove, PA) at room temperature for 1 hour. The proteins of interest were visualized by the luminescence imaging system LAS-4000 (Fujifilm, Tokyo, Japan) with the ECL chemiluminescent detection system (Millipore). β-actin was used as an internal control, and all the proteins were normalized to their own β-actin before compared with that of the control. ImageJ (National Institutes of Health, Bethesda, MD) was used for the quantitative analysis. The experiments were repeated more than three times.

### Flow cytometric analysis

The intensity of tomato fluorescent protein (*pGBeLT*-transduced CD8+ T cells) or expression of intracellular IFN-γ (Biolegend, San Diego, CA) was detected to assess CD8+ T cells activation. To understand the effect of sorafenib on the expression of TGF-β receptor I and FasL, E.G7 cells were incubated with TGF-β receptor I antibody (Abcam), followed by DyLight 488-conjugated anti-rabbit IgG antibody (Kirkegaard & Perry Laboratories, Inc., Gaithersburg, MD), and FasL-PE antibody (eBioscience, San Diego, CA), respectively. For the assessment of Tregs and MDSCs *in vivo*, the tumor draining lymph nodes (TDLNs) and bone marrow were isolated from sorafenib- and vehicle-treated mice (n = 3 per group at each time point). Cells isolated from TDLNs and bone marrow were labeled with anti-FOXP3-Alexa Fluor 488/CD4-APC/CD25-PE antibodies using a Mouse Treg Flow Kit (Biolegend) according to manufacturer's manuals, and CD11b-FITC (eBioscience) and Gr-1-PE (eBioscience), respectively. The fluorescence intensities of these markers were collected using FACSCalibur flow cytometer (BD, San Jose, CA), and analyzed by FlowJo software (Tree Star, Ashland, OR).

### Sorafenib treatment and adoptive cell transfer in E.G7-bearing mouse model

2×10^6^ E.G7 cells were implanted subcutaneously into C57BL/6 male mice. When the average tumor size reached about 100 mm^3^, mice were randomly divided into five groups (n = 4–5 per group): vehicle (control), sorafenib alone (sora), 2×10^6^ CD8+ T cells (2T), 5×10^6^ CD8+ T cells (5T), and 2×10^6^ CD8+ T cells plus sorafenib (2T+sora). For monotherapy, mice were treated with 7.5 mg/kg/day sorafenib alone by gavage for five days, 2×10^6^ CD8+ T cells and 5×10^6^ CD8+ T cells *via i.v.* injection, respectively. For the combination therapy, 2×10^6^ CD8+ T cells were injected one day after the first sorafenib treatment. The same volume of vehicle was given to the control mice for five days. Tumor volumes were measured with a caliper and calculated by the formula: length × width^2^ × 0.523. The experiments were repeated five times.

### Bioluminescence imaging

Mice receiving *pGBeLT*-transduced CD8+ T cells (n = 4 per group) were given 150 mg/kg D-luciferin *i.p*. 15 minutes before imaging. The images were acquired for 5 minutes using the IVIS50 Imaging System (Xenogen, Alameda, CA), and the signals were quantified as photons/s/cm^2^/sr using the Living Image software (Version 2.20, Xenogen).

### Statistical analysis

All data were shown as the mean ± standard error. Student's *t*-test was used for the comparison between two groups. One-way ANOVA followed by Tukey's post hoc test was used when comparing more than two groups. Differences between the means were considered significant if *p*<0.05 or less.

## Results

### Sorafenib inhibits the expression of multiple immunosuppressive factors and death receptor ligand in tumor cells

**Figure 1:**
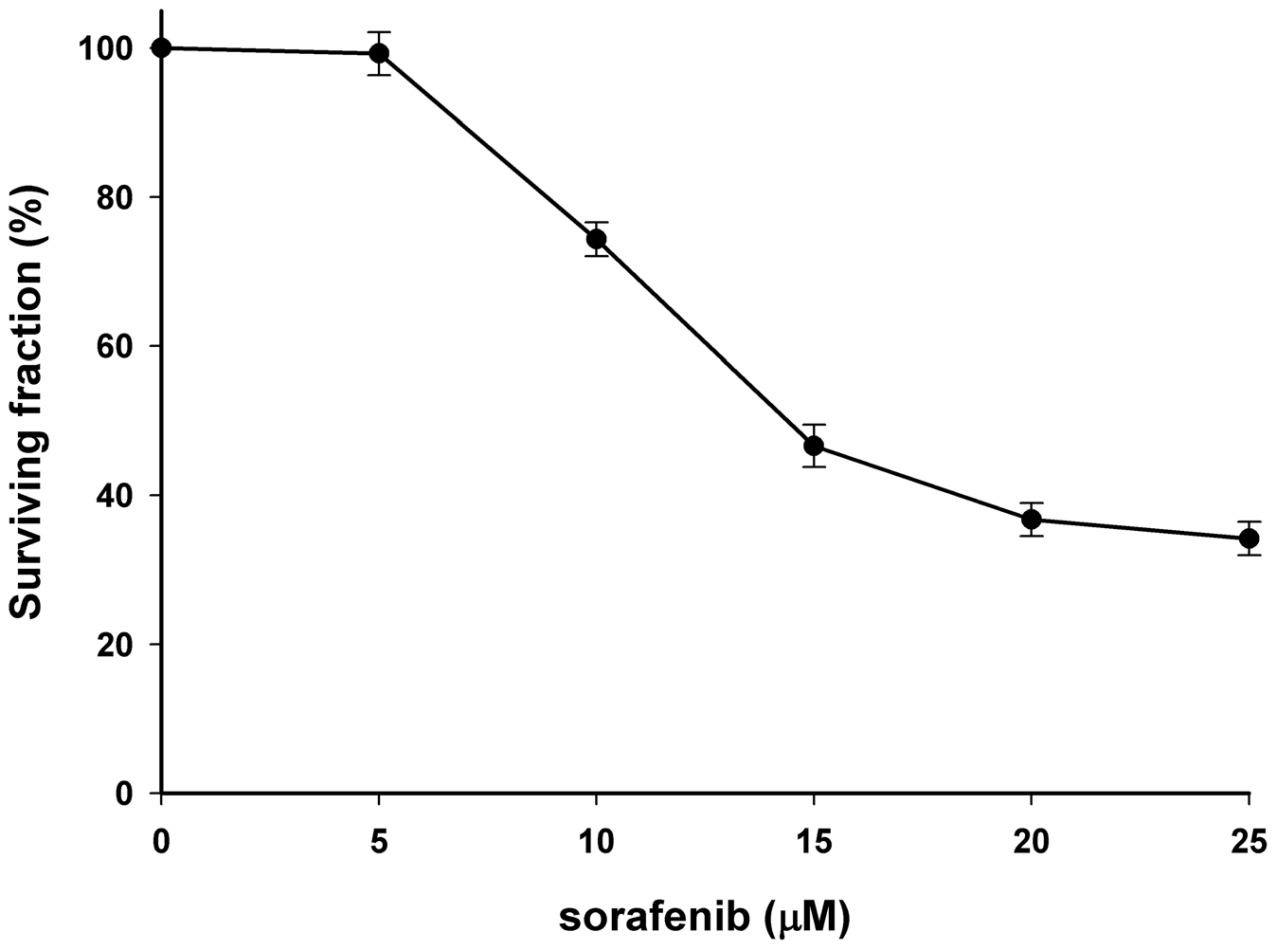
Cytotoxicity of sorafenib on E.G7 cells. E.G7 cells were treated with various concentrations (0–25 µM) of sorafenib for 24 hours. AlamarBlue assay was performed to determine the cell viability. The IC50 of sorafenib is 15 µM on E.G7 cells.

**Figure 2:**
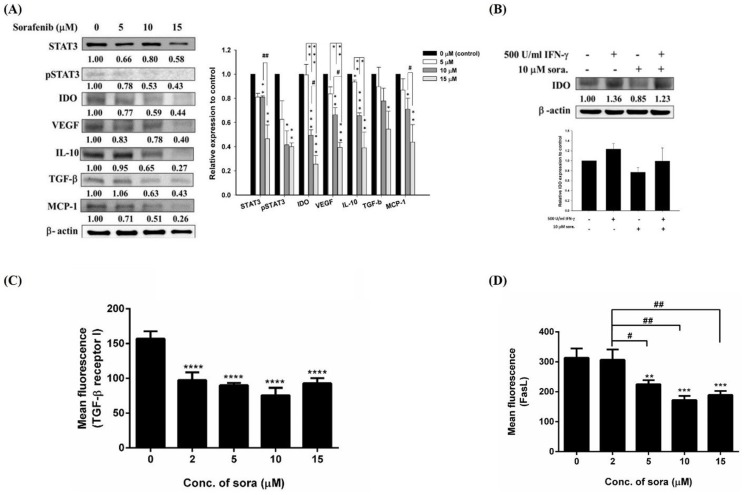
Restoration of immunosuppressive factors in E.G7 cells by sorafenib. (A) (Left panel) Expressions of immunosuppressive factors were assayed by Western blotting, and (right panel) their quantitative results. IDO, VEGF, IL-10 and TGF-βwere decreased in a dose-dependent manner in E.G7 cells treated with 5–15 µM sorafenib for 24 hours. In addition, expressions of pSTAT3 and CCL2/MCP-1 also were decreased. (B) Expression of IDO in E.G7 cells treated with exogenous IFN-γto mimic the tumor-bearing animal model. Sorafenib decreases both endogenous and exogenous IDO expressions. (C) Expression of TGF-βreceptor I was assessed by flow cytometry. TGF-βreceptor I was significant decreased after 2–15 µM sorafenib treatments. (D) Survival of CD8+ T cells would affect the therapeutic outcome of ACT, and apoptosis of CD8+ T cells is mainly through Fas/Fas ligand (FasL) pathway. Thus, expression of FasL was assayed by flow cytometry, and the expression of FasL was suppressed in E.G7 cells by sorafenib. The experiments were repeated more than three times, and one of the representative was shown here. (* as compared with that of vehicle, *p<0.05, **p<0.01, ***p<0.001; ^#^ as compared with that of 2 µM sorafenib treatment, ^#^p<0.05, ^##^p<0.01)

Cytotoxicity of sorafenib was determined by AlamarBlue assay (Figure 1), and the IC50 of sorafenib on E.G7 cells was 15 µM in the following experiments, the doses of sorafenib used were <15 µM (i.e. lower than IC50). To understand whether sorafenib could enhance treatment outcomes of ACT, expressions of well-known immunosuppressive factors including IDO, VEGF, IL-10, TGF-β, and CCL2/MCP-1 in E.G7 cells were evaluated by Western blotting. Sorafenib treatment decreased expressions of these immunosuppressive factors in a dose-dependent manner (Figure 2A). Another immunosuppressive factor, IDO, converts tryptophan to kynurenine resulting in apoptosis of T cells [20]. Except for endogenous expression, IDO could be exogenously induced by IFN-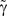. Thus, E.G7 cells stimulated with IFN-γ were treated with sorafenib, and the level of IDO was evaluated by Western blotting. Figure 2B shows that sorafenib inhibited both endogenous and exogenous IDO expressions. TGF-β signaling pathway starts from the binding of TGF-β with TGF-β I and II heterodimer receptors, and the autocrine TGF-β is highly related to endothelial-mesenchymal transformation (EMT) and metastases of cancer [21]. Accordingly, the level of TGF-β receptor I was also assessed by flow cytometry to understand how sorafenib affects TGF-β signaling pathway. As inhibiting of TGF-β, the level of TGF-β receptor I was significantly decreased after sorafenib treatment in a dose-dependent manner (Figure 2C). Fas/FasL signaling pathway triggers apoptosis of T cells, besides tryptophan deprivation by IDO. Interestingly, expression of FasL on the surface of E.G7 cells was significantly lowered by sorafenib in a dose-dependent manner (Figure 2D).

### Sorafenib enhances the activation, killing effects and migratory abilities of CD8+ T cells

**Figure 3:**
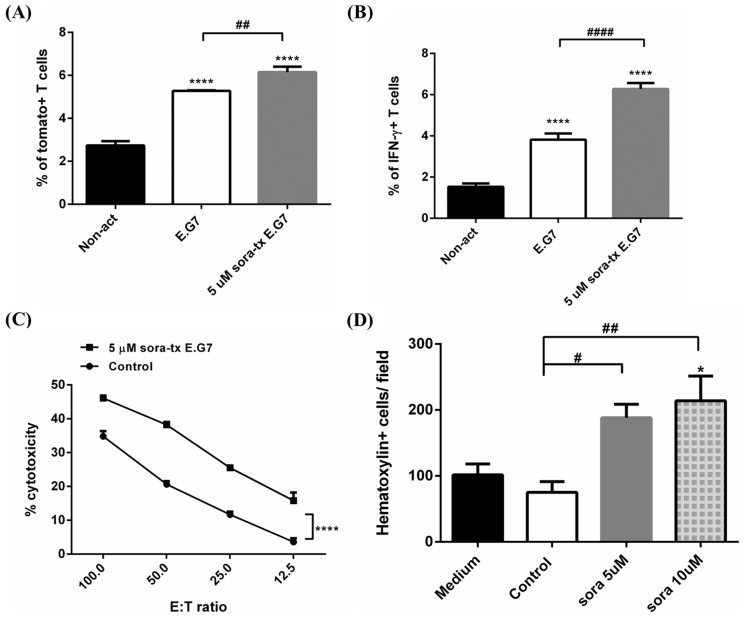
Sorafenib enhances functions and migrations of CD8+ T cells. (A, B) Activities of CD8+ T cells were evaluated from expressions of tomato fluorescent protein and intracellular IFN-γby flow cytometry. Both were elevated after co-cultured with sorafenib-treated E.G7 cells. The experiments were repeated three times, and one of the representative was shown here. (C) Cytotoxicities of CD8+ T cells were enhanced when co-cultured with sorafenib-treated E.G7 cells at different effector-to-target (E:T) ratio. (D) Migratory capability of CD8+ T cells was determined by transwell assay. Sorafenib significantly increased migrations of CD8+ T cells. The experiments were repeated twice. (* p<0.05, ** p<0.01, **** p<0.0001)

To further validate whether the activation of CD8+ T cells could be enhanced by sorafenib treatment, *pGBeLT*-transduced CD8+ T cells were co-cultured with 5 µM sorafenib-treated E.G7 cells, then the expressions of tomato fluorescent protein and intracellular IFN-γ were assessed by flow cytometry. Both numbers of tomato fluorescent protein-expressing and IFN-γ- producing CD8+ T cells were significantly increased (*p*<0.001) (Figure 3A, B). The killing effects of CD8+ T cells on sorafenib-treated E.G7 cells was determined by ^51^Cr-release assay, and the cytotoxicity of T cells was found significantly increased after sorafenib treatment (Figure 3C). Numbers of tumor-infiltrating CD8+ T cells are another key to better ACT outcomes, thus, the migratory ability of CD8+ T cells was determined by transwell assay. Compared with both medium- and untreated controls, CD8+ T cells co-cultured with sorafenib-treated E.G7 cells showed significantly better migratory ability (Figure 3D).

### Sorafenib mediates the immunomodulation through the inhibition of STAT3

**Figure 4:**
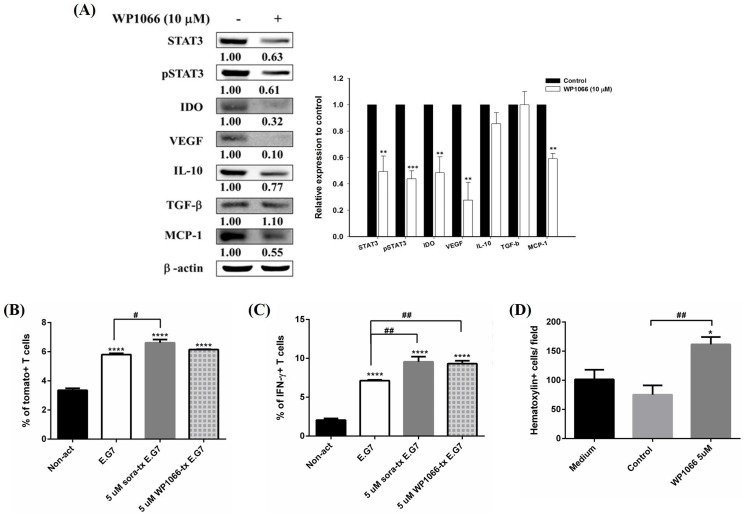
WP1066, an STAT3-specific inhibitor, was used to clarify the role of STAT3 in immunosuppressive microenvironment. (A) (Left panel) Expressions of IDO, VEGF, IL-10, and CCL2/MCP-1 were assayed with Western blotting, and (right panel) their quantitative results. All proteins were suppressed after treated with WP1066. (B, C) Expressions of tomato fluorescent protein and intracellular IFN-γwere assayed by flow cytometry. Significantly higher tomato fluorescent protein and IFN-γsignals were found in CD8+ T cells co-cultured with sorafenib and WP1066-treated E.G7 cells, respectively. (D) Effect of WP1066 on migration of CD8+ T cells was evaluated with transwell assay. More migratory CD8+ T cells were found in WP1066-treated E.G7 cells. The experiments were repeated three times, and one of the representative was shown here. (* p<0.05, ** p<0.01, ***p<0.001, **** p<0.0001)

STAT3 plays a critical role in mediating the antitumor effect of sorafenib on hepatocellular carcinoma [22]. Inhibition of STAT3 with sunitinib, another TKI, has been shown to improve the function of CD8+ T cells in ACT [23]. To verify that sorafenib downregulating immunosuppressive factors shown in Figure 2A is related to STAT3 signaling pathway, the STAT3 inhibitor, WP1066 was used as a positive control. The results showed that STAT3 inhibition downregulated the expressions of IDO, VEGF, IL-10 and CCL2/MCP-1, which also examined in sorafenib-treated E.G7 cells (Figure 4A). Furthermore, *pGBeLT*-transduced CD8+ T cells were co-cultured with WP1066-treated E.G7 cells, determined by flow cytometry and transwell assay. The percentages of both tomato fluorescent protein and intracellular IFN-γ expressions were increased significantly in the WP1066-treated group as compared with the non-treated one (Figure 4B, C). The number of migrated T cells was increased in the WP1066-treated group as well (Figure 4D).

### Serial low doses of sorafenib enhance the therapeutic efficacy of adoptively transferred CD8+ T cells *in vivo*

**Figure 5:**
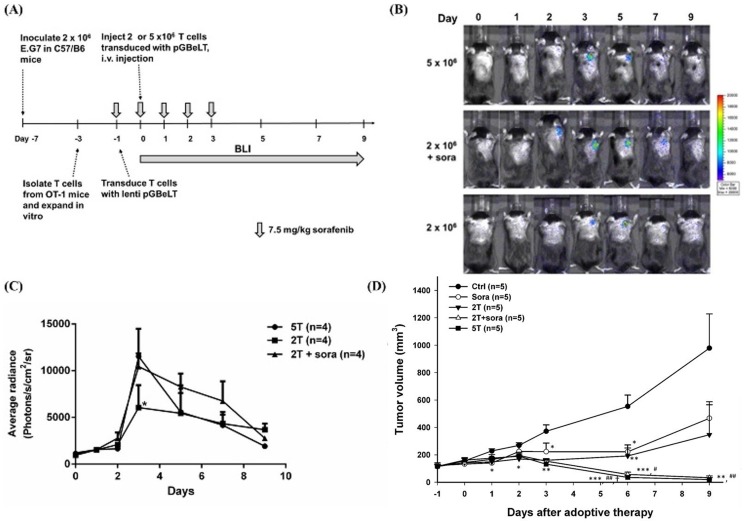
Serial low doses of sorafenib augment recruitment and activation of transferred CD8+ T cells, and exhibit better tumor responses of ACT. (A) The experimental design *in vivo*. (B) Bioluminescent imaging was used to monitor the activation of CD8+ T cells from day 0 post ACT. Transferred CD8+ T cells survived longer in tumor lesions in 2T+sora group compared with that of CD8+ T cells alone group. (C) Quantification of BLI signals from tumors. No significant difference was found between 2T+ sora and 5T groups. Though no significant difference was found between 2T+ sora and 2T groups, about two folds higher average radiance was shown in 2T+ sora group as compared with that of 2T group. (D) Tumor growth was tracked by caliper measurement. Significant tumor shrinkage was observed in 5T and 2T+ sora groups, and no significant difference was found between these two groups. Shrinkage of tumors initiated from day 3 after ACT. Notably, day 3 was the time point with peak BLI signals. The experiments were repeated five times, and one of the representative was shown here. (*as compared with that of the control group, * p<0.05, ** p<0.01, **** p<0.0001; # as compared with that of the sorafenib group, ^#^p<0.05, ^##^p<0.01; †as compared with that of the 2T group, †p<0.05)

**Figure 6:**
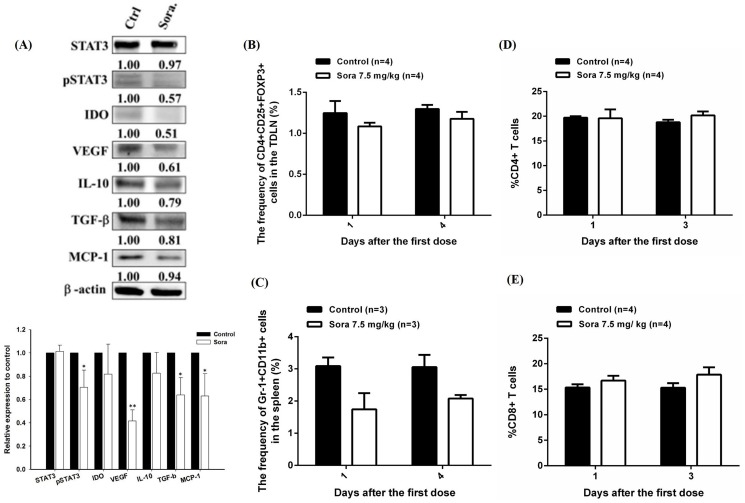
Sorafenib ameliorates therapeutic efficacy of ACT *via* reversing the unfavorable microenvironment. (A) (Upper panel) Immunosuppressive factors in tumors treated with or without sorafenib were evaluated with *ex vivo* Western blotting, and (lower panel) the results were quantified. Immunosuppressive factors were downregulated after sorafenib treatment *via* STAT3 and TGF-β inhibitions. (B, C) Percentages of Tregs in tumor drainage lymph node (TDLN) and MDSCs in the spleen, respectively, were decreased after serial low doses (7.5 mg/kg/day) of sorafenib treatment. (D, E) Percentages of host CD4+ and CD8+ T cells remained unchanged after serial low doses of sorafenib treatments.

When tumor sizes reached 100 mm^3^, E.G7 tumor-bearing mice were randomly divided into five groups: sorafenib alone (sora), 2×10^6^ CD8+ T cells (2T), 5×10^6^ CD8+ T cells (5T), 2×10^6^ CD8+ T cells plus sorafenib (2T+sora), and vehicle control, to determine whether serial low doses of sorafenib could increase the accumulation and activation of transferred CD8+ T cells in target tumors *in vivo*. Mice were received sorafenib by gavage one day before ACT followed by four consecutive doses; the activation of transferred *pGBeLT*-transduced CD8+ T cells and tumor growth monitoring by BLI and caliper measurement were performed, respectively (Figure 5A). The signal of *pGBeLT*-transduced CD8+ T cells was slightly increased from day 0 to day 2, and reached its peak on day 3 in all groups with CD8+ T cells (Figure 5B, C). No significant difference of signal intensity was found between 5T and 2T+sora groups, indicating that sorafenib enhanced the recruitment and activation of CD8+ T cells in target tumors. Sorafenib alone did not show significant tumor inhibition, and 2T group exhibited moderate tumor inhibition (Figure 5D). However, both 2T+sora and 5T groups showed similar tumor inhibitions with substantial tumor regression (Figure 5D). To further investigate whether sorafenib affects the expressions of immunosuppressive in the tumor microenvironment as *in vitro*, *ex vivo* assay in mice bearing E.G7 tumors were conducted. Mice were sacrificed on day 5 post sorafenib treatment, the expressions of immunosuppressive proteins in tumors were detected by Western blotting. The expressions of immunosuppressive proteins were found reduced in comparison with control mice (Figure 6A), indicating that immunosuppressive cells in the tumor microenvironment also were suppressed, since TGF-β, IL-10 and IDO were related to the induction of Tregs and MDSCs. Moreover, MDSCs are thought to be another important negative regulator of CD8+ T cells recently [24]; hence, cells were isolated from TDLNs and bone marrow, and evaluated the changes of Tregs and MDSCs, respectively, using flow cytometry. The percentages of both Tregs and MDSCs were decreased on day 3 compared with the vehicle control group (Figures 6B, C). The results showed that sorafenib could downregulate immunosuppressive cell populations, resulting in the improvement of CD8+ T cell function. Most importantly, no significant change was found in CD4+ and CD8+ T cell populations of the host after sorafenib treatment (Figures 6D, E).

## Discussion

Both of preclinical and clinical studies have shown that the tumor microenvironment plays an indispensable role in limiting the effectiveness of ACT. Blocking the key regulatory pathways of immunosuppression in the tumor microenvironment can improve antitumor effects of ACT substantially. In this study, a well-established E.G7/OT-1 murine model was utilized to prove that sorafenib, a multi-kinases targeting drug used in HCC and RCC treatments, can reverse the unfavorable tumor microenvironment and enhance therapeutic efficacies of ACT both *in vitro* and *in vivo*.

The combination of ACT with chemotherapy for cancer treatments is still challenging because of the immunosuppressive effects of most chemotherapeutic drugs. Chemotherapeutic drugs such as paclitaxel (TAX), cisplatin (CIS), and doxorubicin (DOX) have been shown some positive immunological effects which may help outcomes of ACT. These drugs modify the immune microenvironment *via* several mechanisms, including better immunogenicity induced by tumor apoptosis [25], [26], upregulations of tumor-associated antigens and mannose-6-phosphate (M6P) expressions and modulation of Fas/TRAIL-dependent pathway sensitize tumor cells to cytotoxic T lymphocytes (CTLs) [27]–[29], elimination of Tregs [30], [31], and disruption of tumor stromal caused larger numbers of tumor-infiltrating CTLs [32]. Sorafenib downregulates the expression of TGF-βin E.G7 cells, and other immunosuppressive factors, such as IDO, VEGF and IL-10 (Figures 2A, B). Not only TGF-β expression was decreased, but fewer TGF-β I receptors on cell surfaces were found after sorafenib treatment (Figure 2C), suggesting that sorafenib blocks the TGF-β signaling pathway, which is critical for tumor progression and immunosuppression. IDO, VEGF and IL-10 have been reported to lower the activities of CD8+ T cells, increase the conversion of Tregs from CD4+ T cells and polarize macrophages into immunosuppressive M2 subtype [2], [3]. On the other hand, the number of tumor-infiltrating lymphocytes (TILs) also contributes to the result of ACT [33]. Angiogenesis is highly related to infiltration of CD8+ T cells and regulation of other immunosuppressive cells [34], [35], and sorafenib has been shown to target the expression of VEGFR in the endothelial cells [11] and reduce the expression of VEGF in cancer cells [18]. Moreover, CCL2/MCP-1, acting as a monocyte chemoattractant and immunosuppressive factor, inhibits CD8+ T cells infiltration through the recruitment of TAMs and Tregs [36]. CCL2/MCP-1 plays a key role for immunosuppression, so that inhibition of CCL2/CCR2 pathway could be a possible approach for cancer treatment [37], [38]. Prolonging the survival of CD8+ T cells has been proofed related to outcome of ACT by transduced with Bcl-2, an antiapoptotic gene [39]. FasL plays an influential role in immunosuppression by sensitizing activated CD8+ T cells to Fas-mediated apoptosis pathway [40]. Ryan et al. demonstrated that blocking FasL expression would suppress the immune escape *in vivo* by using FasL^-/low^ cell line [41]. As shown in Figure 2D, the FasL expression of E.G7 was decreased after sorafenib treatment, which may inhibit Fas/FasL-mediated apoptosis in CD8+ T cells.

Furthermore, the killing effects, activation and migratory abilities of CD8+ T cells were measured by flow cytometry, ^51^Cr-release and transwell assays. CD8+ T cells co-cultured with sorafenib-treated E.G7 cells were shown with higher killing effects, activation, and migratory abilities as compared to those co-cultured with untreated E.G7 cells (Figure 3), suggesting that the suppressions of immunosuppressive factors after sorafenib treatment should play a role in tumor microenvironment (Figure 2). STAT3 is an important regulator of immunosuppression [42]. For instance, TGF-β VEGF and IL-10 expressions are regulated by STAT3; furthermore, these proteins insure the continuance of STAT3 activation in immunosuppressive tumor microenvironment. Thus, inhibiting STAT3 in cancer cells would be a novel approach to reverse immunosuppression and improve results of immunotherapies. Kujawski et al. verified the impact of STAT3 on ACT and showed the restoration of T cell activity and better tumor inhibition in STAT3^-/-^ mice [23]. In their study, another TKI, sunitinib was used to combine with ACT. Phospho-STAT3 (pSTAT3) expressed in tumor cells was inhibited by sunitinib treatment. Moreover, sunitinib treatment resulted in significant tumor growth inhibition, elevated numbers of TILs and decreased Tregs accumulation in tumors. Sorafenib has been proved to downregulate STAT3 pathway in several types of cancer [22], [43], and similar results were also found in this study. Moreover, WP1066, an STAT3 inhibitor, was used as a positive control in this study to verify the lower expressions of those immunosuppressive factors related to downregulation of STAT3 signaling pathway (Figure 4). Interestingly, the expression of TGF-β did not change in E.G7 cells after WP1066 treatment. However, sorafenib modifies the tumor microenvironment not only through STAT3 pathway, but TGF-β pathway as well. Whether sorafenib improves the tumor microenvironment to enhance the function of OT-1 CD8+ T cells through other signal pathways other than STAT3 pathway needs to be further investigated.

To investigate how sorafenib augments functions of CD8+ T cells *in vivo*, we applied five consecutive low doses of sorafenib (7.5 mg/kg/day) to a well-established E.G7/OT-1 murine model for understanding the immune modulations and clarifying whether this approach could be applied in ACT. The numbers of CD8+ T cells for combination therapy was determined according to our preliminary study. Various numbers of CD8+ T cells were transferred into E.G7 tumor-bearing mice, the tumors grew again from day 9 after adoptive transfer when two million or less CD8+ T cells were transferred. On the contrary, complete tumor inhibition was found in mice received 5×10^6^ or 10×10^6^ CD8+ T cells (Figure S1). Thus, 2×10^6^ CD8+ T cells (2T) were used for combination with low doses of sorafenib. As shown in Figure 5C, the activation of CD8+ T cells in 2T+sora group was similar to that of 5T group and was significantly better than that of 2T alone group. The peak signals obtained from these two groups were detected on day 3 post ACT, but the signal of the 2T+sora group existed longer than that of 5T group. Notably, tumors started to shrink from day 3 in both 5T and 2T+sora groups (Figure 5D) with no significant difference between these two groups, suggesting that the combination of smaller numbers of CD8+ T cells with serial low doses of sorafenib as a potential therapeutic strategy for cancer treatment. In addition, sorafenib decreased STAT3 and related immunosuppressive factors after serial low doses of sorafenib treatment as shown by Western blotting (Figure 6A). Some of these proteins are thought to be related to the expansion of Tregs and MDSCs, both could result in the inhibition of CD8+ T cells. Here we found that these two immunosuppressive cells were decreased after serial low doses of sorafenib treatment (Figures 6B, C). These findings are similar to those reported by Cao et al. in a murine liver cancer model [19], in which the percentages of Tregs and MDSCs were significantly decreased after tumor-bearing mice were treated with 30 mg/kg/day for two weeks. Although both types of cells were decreased, no significance was found in our study. The possible reasons responsible for this difference may be due to the low dose, i.e. 7.5 mg/kg/day (one fourth of the dose used by Cao et al.), and the shorter treatment time (five days) were used in this study. Recently, Chen et al. also showed that sorafenib decreased Tregs population and PD-1 expressions on CD8+ T cells in a HCC-bearing animal model [44], which were similar to our findings.

The usual dose of sorafenib used for the treatments of advanced HCC, RCC and thyroid cancer is 400 mgx2/day [45]–[47], however, adverse side effects are reported including hand-foot syndrome [48], [49], skin tumors [50] and small intestine hemorrhage [51]. The dosage used in mice can be converted into the human equivalent dose (HED) with the following formula: HED (mg/kg)  =  dose in mouse (mg/kg) ×3_(mouse Km)_/37_(human Km)_, in which Km is the body weight divided by the body surface area [52]. The dosage of sorafenib (7.5 mg/kg/day) used here resulted in a HED is only about one twentieth as compared to the usual dose. Doses of sorafenib often used in the mouse model range from 15 to 100 mg/kg/day [18], [19], [53], [54]. Therefore, the 7.5 mg/kg/day of sorafenib is so called as “low dose” in this study, which may avoid the adverse side effects found under the usual dosage.

Hosoi et al. proved that CD8+ T cells themselves recruit MDSCs to tumors by secreting IFN-γ and CCL2/MCP-1, which would further inhibit transferred CD8+ T cells and reduce the outcome of ACT [55]. New strategies to improve the survival and function of transferred CD8+ T cells are needed, to extend the “transient” antitumor effects of regular ACT. Increasing evidences show that sorafenib improves the antitumor effects through both innate and adaptive immunity, including enhancement of the crosstalk between macrophages and NK cells [56], and downregulation of Tregs in RCC patients [13], [14]. On the other hand, BRAF inhibitors have been shown to ameliorate functions of CD8+ T cells through downregulation of VEGF [57] or CCL2 [58] in tumors, suggesting that sorafenib may be helpful in combination with ACT under the optimal dose and suitable timing since sorafenib is also a BRAF inhibitor.

**Figure 7:**
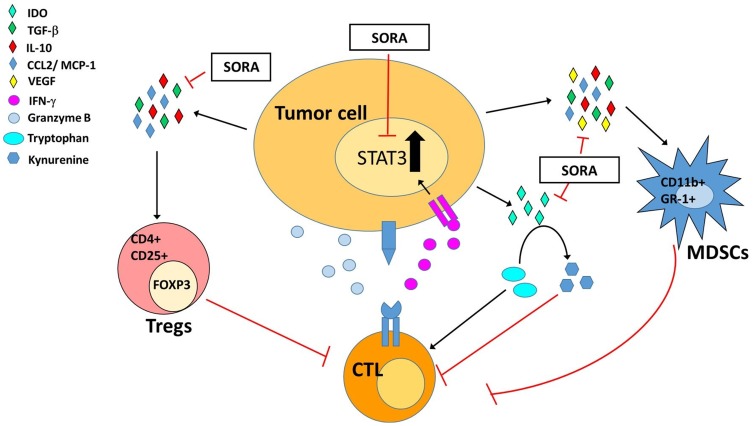
Sorafenib augments the therapeutic efficacy of ACT by inhibiting STAT3 expression and downregulating immunosuppressive factors in tumor cells and tumor microenvironment. Downregulation of immunosuppressive factors, including TGF-β, IL-10, CCL2/MCP-1 and VEGF, lowers the populations of both Tregs and MDSCs. Transferred CD8+ T cells show better activation and killing effects due to declined Tregs and MDSCs. Furthermore, more transferred CD8+ T cells accumulate in tumors and survive longer, so that enhanced therapeutic responses can be achieved.

Our findings may pave the way for clinical ACT studies in patients with metastatic renal cell cancer or hepatocellular carcinoma who will receive sorafenib as a standard care. Once phase I clinical studies in this particular cancer entity have been successfully conducted, one might even think of administering low-dose sorafenib as an enhancer of immune therapies such as ACT or other in different cancer entities, including melanoma or multiple myeloma. In the future, it will be worth to discover other immunomodulatory agents as sorafenib to be combined with ACT or other immune therapies, and the effects of low-dose sorafenib on other immune cells, such as DCs and TAMs, in ACT, to further elucidate the panorama of immune system and the tumor microenvironment in ACT. In conclusion, low doses of sorafenib may be an ideal immunomodulatory agent for improving the therapeutic outcome of ACT through downregulation of immunosuppressive factors and enhancement of T cells functions *via* inhibitions of STAT3. Inhibitions of these immunosuppressive factors decrease the conversion of Tregs and induction of MDSCs, which otherwise will impede the function of CD8+ T cells. IDO, responsible for tryptophan metabolism in tumor cells, is decreased both endogenous and exogenous after sorafenib treatment. Transferred CD8+ T cells may exhibit better functions and survive longer and improve the therapeutic efficacy of ACT when these immunosuppressive factors and cells decline Figure 7. Furthermore, administration of low doses of sorafenib prior to ACT could reduce the numbers of transferred CD8+ T cells, which is one of obstacles hindering ACT applications. Using this combinational strategy would facilitate the employment of ACT; on the other hand, the lower doses of sorafenib and CD8+ T cells may also improve the quality of life of patients.

## Supporting Information

Figure S1 **Inhibitions of E.G7 tumors by various numbers of transferred OT-1 CD8+ T cells.** 1×10^6^ (1T) to 10×10^6^ (10T) OT-1 CD8+ T cells were transferred via *i.v.* injection into E.G7 tumor bearing mice when the tumor sizes reached 100 mm^3^. Tumor sizes were monitored by caliper measurement. (*as compared with that of the control group, *p<0.05).
(TIF)

Checklist S1 **A checklist uses to ensure that the study was designed properly and the animal experiments were performed reasonably.**
(DOC)
