# Supplementary figures and images for: Serial Low Doses of Sorafenib Enhance Therapeutic Efficacy of Adoptive T Cell Therapy in a Murine Model by Improving Tumor Microenvironment
Source: PLoS One. 2014 Oct 15;9(10):e109992. doi: 10.1371/journal.pone.0109992 (PMC4198194; doi:10.1371/journal.pone.0109992)

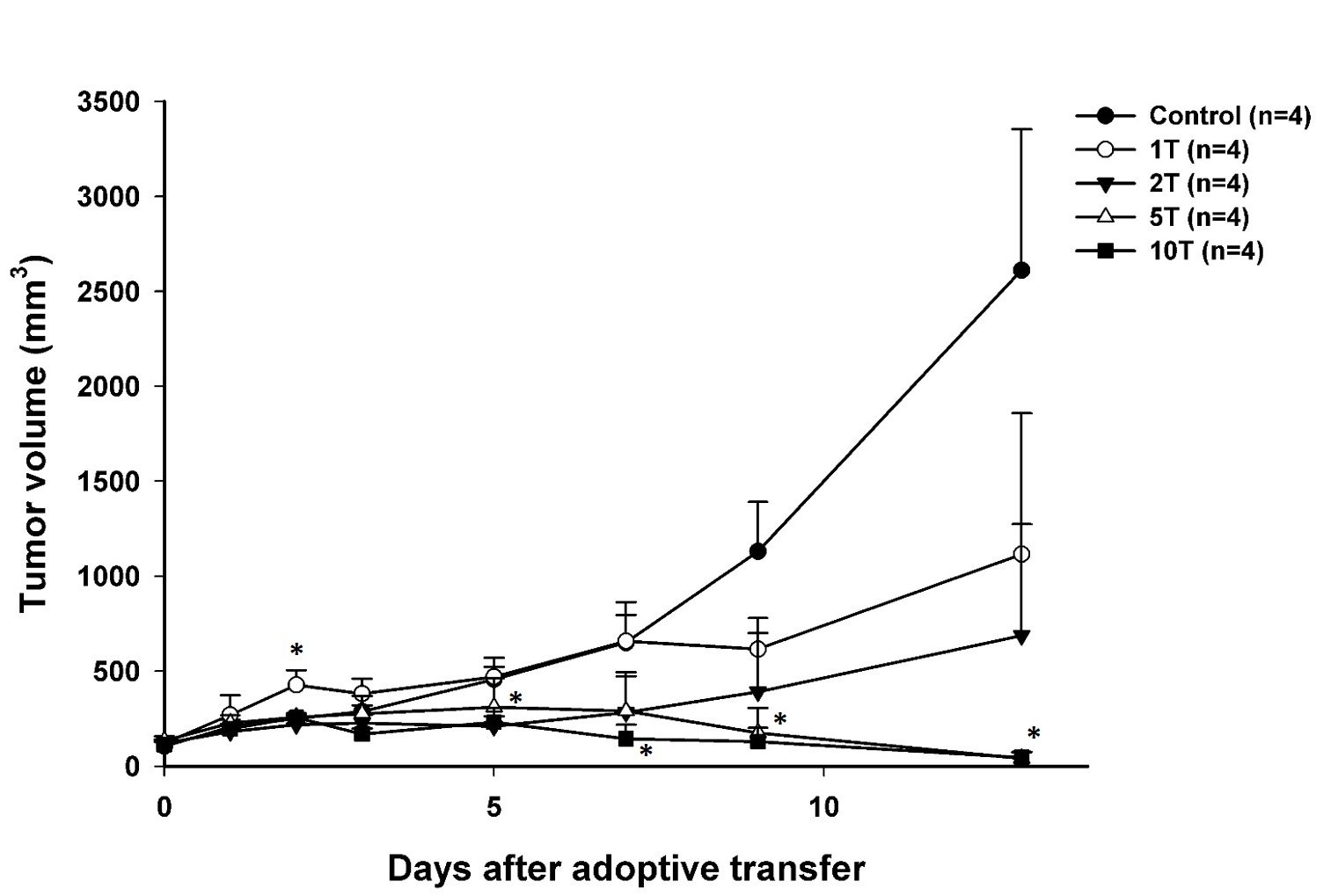

Supplement: Figure S1 — Inhibitions of E.G7 tumors by various numbers of transferred OT-1 CD8+ T cells. 1×10^6^ (1T) to 10×106 (10T) OT-1 CD8+ T cells were transferred via i.v. injection into E.G7 tumor bearing mice when the tumor sizes reached 100 mm3. Tumor sizes were monitored by caliper measurement. (*as compared with that of the control group, *p<0.05). (TIF) [file pone.0109992.s001.tif]
